# Epidemic of an Urticarioid Dermatitis Due to a Small Mite (*Pediculoides Ventricosus*) in the Straw Mattresses

**Published:** 1909-09

**Authors:** Joseph Goldberger, Jay F. Schamberg

**Affiliations:** Passed Assistant Surgeon, U. S. Public Health and Marine-Hospital Service; Professor of Dermatology and Infectious Eruptive Diseases in the Philadelphia Polyclinic.


### SELECTED ARTICLE

### Epidemic of an Urticarioid Dermatitis Due to a Small Mite (*Pediculoides ventricosus*) in the Straw Mattresses.

A preliminary report by JOSEPH GOLDBERGER, Passed Assistant Surgeon, U. S. Public Health and Marine-Hospital Service, and JAY F. SCHAMBERG, Professor of Dermatology and Infectious Eruptive Diseases in the Philadelphia Polyclinic.

[Reports to the Surgeon-General, Public Health and Marine-Hospital Service.]

(*Public Health Reports*, July, 1909.)

WE wish to invite the attention of the profession to a skin affection of unusual character which has prevailed in an epidemic form in Philadelphia and vicinity since the early part of May, 1909. We have reason to believe that this disease is not confined to the locality indicated, but occurs more or less in various parts of the United States.

In 1901, Schamberg published (*Phil. Med. Jour.,* July 6, 1901,) a short article on "An Epidemic of a Peculiar and Unfamiliar Disease of the Skin," examples of which were in that year simultaneously observed for the first time by Schamberg, Duhring, Hartzell, Stelwagon, and other dermatologists in Philadelphia. Since 1901 cases of this same character have been encountered each year, usually between the months of May and October.

*Etiology.*—The cause of the peculiar affection which we are considering was until recently very obscure. During the months of May and June, 1909, an outbreak (20 cases) of this eruptive disease developed among the crew upon a private yacht docked in the Delaware River. At almost the same time 33 more cases appeared among the crews of 4 other boats. Besides these 53 cases we learned in the course of our investigation of about 70 other cases in 20 different private residences and boarding houses scattered about the city of Philadelphia and its vicinity. In practically every case we were able to determine that the patient had either recently slept upon a new straw mattress or had freely handled the same. The facts elicited by our inquiry enabled us to exclude from consideration the jute or cotton topping or the ticking of the mattresses and we satified ourselves that the essential causative factor was connected with the wheat straw. The mattresses were made by four of the leading manufacturers, all of whom received a large proportion if not quite all of their straw from the same source in New Jersey.

In order to establish the etiological rôle of the straw mattresses experimentally, one of us exposed his (left) bare arm and shoulder for one hour between two straw mattresses. At the end of about sixteen hours the characteristic itching eruption appeared. Later three volunteers slept upon a mattress during a night and each one developed the eruption at the end of about the same period.

We next took some of the straw and sifted such particles as would pass through the meshes of a fine flour sieve. The sifted particles were divided into two portions and placed in two clean glass Petri dishes. One of these was then applied for one hour to the left axilla of a volunteer. At the end of about sixteen or eighteen hours the characteristic eruption was present in the area of the left axilla to which the Petri dish of straw siftings had been applied.

Having therefore determined not only by deduction from the epidemiological facts but by experiment that the straw in the straw mattresses was in some way capable of producing the eruption we next sought in the straw for the responsible factor. First we exposed for an hour the second portion of the siftings in a Petri dish to the vapor of chloroform under a bell jar with a view to killing any insect or acarine that might be present. These siftings were then applied to the right axilla of the volunteer to whose left axilla the untreated siftings were applied. While, as has been stated, the application of the untreated siftings was followed by the appearance of the characteristic eruption, the skin to which the chloroformised siftings were applied remained perfectly normal. We inferred, therefore, that the essential causative factor residing in the straw had been killed by the chloroform fumes. Careful scrutiny of some of the fresh siftings from the straw disclosed the presence of a small almost microscopic mite. Five of these mites were fished out, placed in a clean watch crystal and then applied to the axilla of another volunteer. At the end of about sixteen hours following this application five of the characteristic lesions appeared on the area to which the mites had been applied.

We established, therefore, that the minute mite which we fished out of the straw siftings was the factor in the straw that was responsible for the production of the eruption. This mite was identified for us by Mr. Nathan Banks, expert in acarina of the United States Bureau of Entomology, as very close to, if not identical with, *Pediculoides ventricosus*.

We have encountered the disease only between the months of May and October, in Philadelphia and its vicinity. A patient with this affection was exhibited by one of us before the American Dermatological Association in June, 1909. Prominent dermatologists from Boston, Baltimore, New York, Chicago, St. Louis, San Francisco and London, stated that they were unfamiliar with the clinical picture presented.

*Eruption.*—The disease is characterised, as a rule, by an eruption consisting of wheals, nearly all of which are surmounted by a central vesicle, which very rapidly acquires turbid and later pustular contents. This is the peculiar and characteristic lesion of the affection. Instead of frank wheals, the primary efflorescences may be erythemato-urticarial spots or papulo-urticarial lesions. They vary in size from a lentil seed to a finger nail, and are rounded, oval, or irregular in shape. They are of a warm rose color, but only rarely exhibit the pinkish white anemic area seen in the lesions of ordinary "hives." The central vesicle is usually minute, not exceeding a pin head in size; in other cases it may be larger, acquiring the dimensions of a lentil seed or pea.

The eruption is more or less profuse and usually extends over the neck, chest, abdomen, and back, and in a lesser degree over the arms and thighs. Scattered lesions are often observed on the face, forearms, and legs, but the hands and feet are nearly always free. The extent of the eruption and the size of the individual lesions are apt to bear an inverse proportion to each other. In the most profuse eruptions 10,000 or more lesions may be present. In some cases the eruption described may undergo modification and later present patches conforming to the type of erythema multiforme. There are, therefore, three varieties of eruption (*a*) urticaria vesiculo-pustolosa, (*b*) erythema multiforme, (*c*) varicelloid type with large central vesicle or pustule.

The eruption is accompanied in well-pronounced cases by the most intolerable itching, which for obvious reasons is worse at night and may seriously interfere with sleep. The pruritus may lead to violent scratching with the production of excoriations.

*Systemic Symptoms.*—Some patients with profuse eruptions have an elevation of temperature varying from 99° F. to 102° F. There may also be at times malaise and anorexia, although as a rule patients do not complain of feeling ill and rarely seek their bed. There is, in some patients, a moderate enlargement of the subcutaneous lymph glands. In three instances transient albuminuria was observed.

The affection is apt to be confounded with ordinary "hives" or urticaria, chickenpox and scabies. We have known many such errors of diagnosis to have been made. In one case with a particularly profuse eruption, the patient was under suspicion of suffering from smallpox.

We have received a number of letters from laymen and physicians in Pennsylvania and Ohio alleging that farmers commonly develop a hive-like eruption after contact with oat straw and rye straw, and that these are therefore not used for bedding. There are several references in foreign literature to mites of the genus *Pediculoides* in grains attacking man and producing cutaneous lesions.

*Treatment.*—The mattress may be exposed to sulphur fumes, to steam, or to formaldehyde in a vacuum chamber to kill the mite. For the relief of the itching and the cure of the cutaneous condition the following has been found efficacious:

℞ Betanaphtol ........................gr. xxx

Sulphur. precip........................ʒi

Adipis benzoat ........................℥i

Ordinarily the itching will subside within twelve to thirty-six hours and the eruption will disappear in about a week or ten days. Where, however, the cause is not recognised and the use of the mattress is continued we have known patients to suffer severely for periods of from three to seven weeks, when gradual subsidence and recovery would take place.

We have known patients to be obliged at times to discontinue their daily work owing to loss of sleep and the distress due to itching. Other patients were compelled by their employees to cease work owing to the suspicion of contagion and the opprobium attaching to the presence of a profuse eruption.

A more exhaustive report of this investigation will, it is hoped, be published later.

